# Advanced SEM and TEM Techniques Applied in Mg-Based Hydrogen Storage Research

**DOI:** 10.1155/2018/6057496

**Published:** 2018-07-17

**Authors:** Jianding Li, Jincheng Xu, Bo Li, Liqing He, Huaijun Lin, Hai-Wen Li, Huaiyu Shao

**Affiliations:** ^1^Institute of Applied Physics and Materials Engineering (IAPME), University of Macau, Macau; ^2^Department of Materials Science and Engineering, Southern University of Science and Technology, Shenzhen 518055, China; ^3^Institute of Advanced Wear & Corrosion Resistance and Functional Materials, Jinan University, Guangzhou 510632, China; ^4^Kyushu University Platform of Inter/Transdisciplinary Energy Research (Q-PIT), Kyushu University, Fukuoka 819-0395, Japan

## Abstract

Mg-based materials are regarded as one of the most promising candidates for hydrogen storage. In order to clarify the relationship between the structures and properties as well as to understand the reaction and formation mechanisms, it is beneficial to obtain useful information about the size, morphology, and microstructure of the studied materials. Herein, the use of scanning electron microscopy (SEM) and transmission electron microscopy (TEM) techniques for the representation of Mg-based hydrogen storage materials is described. The basic principles of SEM and TEM are presented and the characterizations of the size, morphology observation, phase and composition determination, and formation and reaction mechanisms clarification of Mg-based hydrogen storage materials are discussed. The applications of advanced SEM and TEM play significant roles in the research and development of the next-generation hydrogen storage materials.

## 1. Introduction

Mg-based materials are thought to be very promising solid state hydrogen storage systems for mobile or stationary applications due to its low price, abundant resources, and high theoretical hydrogenation capacity [1–10]. However, the challenges of poor kinetics and improper thermodynamics seriously hindered their commercial applications. The situation of the little practical use of Mg-based hydrogen storage materials is mainly attributed to the extremely slow hydrogenation/dehydrogenation reaction, which occurs only at high temperatures (above 523 K). In practice, an activation process of absorption/desorption also requires a high temperature of about 623 K and a hydrogen pressure of 70 atm without any additives [11]. It is reported that there are several reasons which may limit the reaction kinetics. The first one is that the magnesium oxide forms easily on the surface of Mg when the Mg particles are exposed to air. Moreover, the formed MgO layer on the surface of Mg would greatly prevent the contact of Mg and hydrogen molecules and hamper hydrogen penetration into the Mg-based materials to form a metal hydride. Another one is that the dissociation rate of hydrogen molecules on the metal surface is slow. Numerous efforts are required to improve the kinetics and to tailor the thermodynamics in Mg-based materials. To overcome these drawbacks, different nanoprocessing techniques are adopted to synthesize Mg-based nanomaterials for hydrogen storage development. These techniques include ball milling, hydrogen plasma metal reaction (HPMR), catalyzed solution chemical synthesis, and nanoconfinement [8, 12–27]. Particularly, nanoprocessing for the synthesis of nanosized Mg-based materials has gained more and more interest because of the need to increase the surface contact between Mg and hydrogen and to reduce the diffusion distance for hydrogen in particles and grains [28–30]. Moreover, it is beneficial to represent the microstructures of Mg-based hydrogen storage materials when researchers want to figure out the relationship between the structure and hydrogen storage performance. Lots of work have been reported on the study of the nanoscale structural characterization of a Mg-based hydrogen storage material by TEM and SEM techniques [31–41]. In some cases, in order to give direct evidence of the operating mechanisms, in situ scanning techniques can be used to directly understand the hydrogen reaction mechanism of MgH_2_ [42–46]. Here, the use of conventional electron microscopies as well as in situ techniques to observe the microstructure information in Mg-based materials and to understand the formation and reaction mechanisms during hydrogen storage processes is described. The main materials discussed in this work include the following:

- Mg and other alloy metal nanoparticles (Ni, Cu, Co, Fe, and Al) synthesized by a hydrogen plasma metal reaction method (size measurements, morphology observation, and correlation study of properties and microstructure)
- Mg_50_Co_50_ alloy with a body-centered cubic (bcc) structure synthesized by ball milling for 100 h (size measurements and phase and composition determination)
- Mg thin-film sample deposited on a glass substrate by a sputtering method (size measurements)
- Commercial 325 mesh Mg from Alfa Aesar (morphology observation)
- Mg_50_Co_50_ samples ball milled for various durations (determination of the phase and composition and understanding the formation mechanism)
- TiH_2_ catalyzed a MgH_2_ nanocrystallite sample by a chemical solution synthesis method (phase and composition determination)
- MgH_2_ and Mg_2_NiH_4_ samples from the hydrogenation of Mg and Mg_2_Ni (in situ observation of hydrogen reaction mechanism)

## 2. Application of SEM and TEM Techniques for Mg-Based Hydrogen Storage Research

### 2.1. Size Measurements (Particle Size, Crystallite Size, Etc.)

With the emerging of more and more nanotechnologies in the development of Mg-based hydrogen storage materials, it is important to obtain the size information in nanometer and micrometer scales, so that we may understand more about the size effect on hydrogen storage properties in these materials. There have been numerous analytic techniques for size measurements, such as electron microscopy, dynamic light scattering, X-ray diffraction and scattering, field flow fractionation, centrifugal liquid sedimentation, and atomic force microscopy. The TEM technique may provide a two-dimensional picture of sample particles, which can be used for the size distribution evaluation in certain area ranges [47].

**Figure 1:**
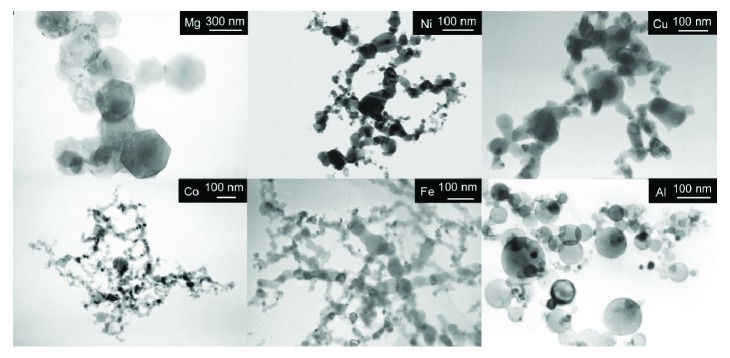
TEM images of different metal nanoparticles synthesized by the hydrogen plasma metal reaction method (Mg, Ni, Cu, Co, Fe, and Al) (reproduced with permission from [8]).

Figure 1 shows the TEM images of Mg, Ni, Cu, Co, Fe, and Al nanoparticle samples obtained by a hydrogen plasma metal reaction method. All of these metal nanoparticles after HPMR synthesis show a granular structure. The particle sizes of these nanoparticle samples for various metals are quite different. Mg particles have a much larger size than the others. The average size of Mg particles is around 300–500 nm, while the ones for Ni, Cu, Co, Fe, and Al are around 30–50 nm. The difference is due to the much faster vaporization rate and higher synthesis rate of Mg than the other metals during the HPMR synthesis process. The evaporation rate depends much on the vaporized metals and it is strongly related to the melting points, boiling points, and saturation vapor pressures of these metals, which influence the size of the synthesized metal nanoparticles [48]. In Figure 1, we can see that the size and distribution of the particles may be easily obtained from TEM observation.

**Figure 2:**
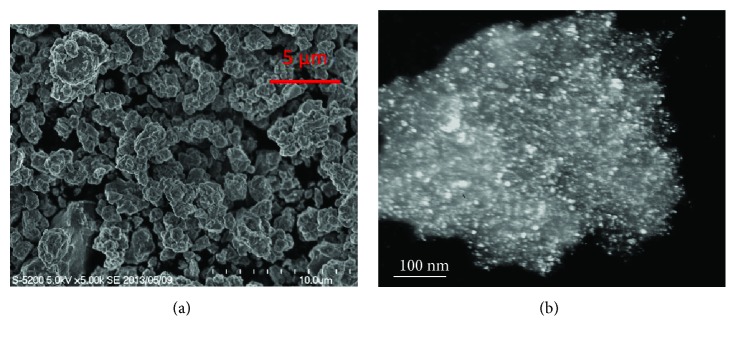
(a) SEM image and (b) dark-field TEM image of the Mg_50_Co_50_ alloy with a body-centered cubic structure synthesized by ball milling for 100 h (reproduced with permission from [50]).

Figure 2 presents the SEM image and dark-field TEM image of the Mg_50_Co_50_ alloy with a bcc structure. This alloy with a metastable feature and unique microstructure was reported to be able to absorb hydrogen at −15°C, which is the lowest temperature reported so far for Mg-based materials to absorb hydrogen [8, 49]. The microstructure information of this sample can be obtained by SEM and TEM observations. From Figure 2(a), we can see that the Mg_50_Co_50_alloy after ball milling has a homogenous particle size of around 1-2 *μ*m. From the dark-field TEM in Figure 2(b), we may find some crystallites inside a large particle of the Mg_50_Co_50_ alloy with a size of just a few nm, which is in agreement with the crystallite size calculated by the broadening of X-ray diffraction peaks [50–52]. The very fine particle size of 1-2 *μ*m, the crystallite size of a few nm, and the bcc nonclose structure are thought to be the main factors of good kinetics at low temperature. In Figures 2(a) and 2(b), we can see that SEM focuses on the surface of the sample, while TEM may see more inside the particle or beyond the surfaces, which makes it possible to measure particle size by SEM observation on the surface and obtain crystallite size inside the particles via transmitted electrons illuminated in the image.

**Figure 3:**
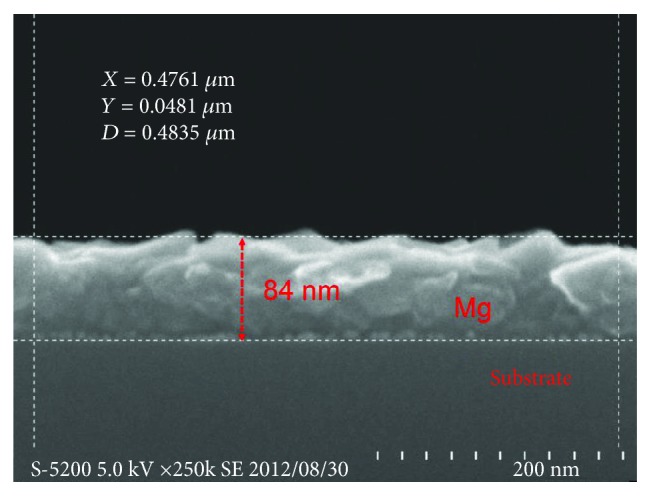
SEM image of the cross-sectional observation of the Mg thin film on a glass substrate (reproduced with permission from [50]).

In Figure 3, we use the SEM technique to obtain the thickness size of the thin film. This size is needed for the calculation of thermal diffusivity and thermal conductivity of thin-film samples. From Figure 3, the boundary of the Mg film with the glass substrate is clear and it can be clearly observed that the layer thickness is about 84 nm. With this value, the thermal diffusivity and thermal conductivity of the Pd-capped Mg thin film are calculated to be 4.62^∗^10^−5^ m^2^/s and 82.0 W/m/K, respectively. When we compare the thermal conductivity and hydrogen absorption kinetics with several other Mg-based materials (325 mesh Mg, Mg single crystal (0001), Mg nanoparticles by HPMR method, and Mg_50_Co_50_ bcc alloy) the Mg thin-film sample is the optimized sample with superior hydrogen absorption kinetics and good thermal conductivity. By SEM observation, the key thickness size of the Mg thin film is obtained and this makes it possible for the evaluation of thermal physical properties.

### 2.2. Morphology Observation

**Figure 4:**
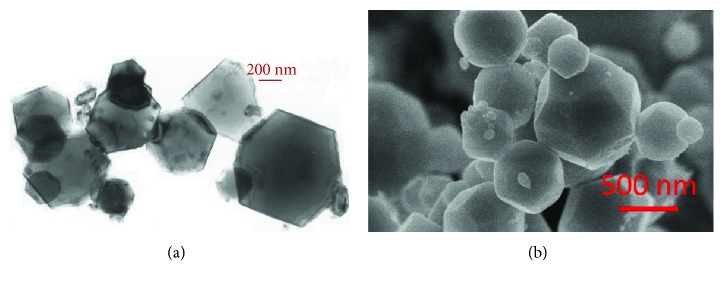
(a) TEM image and (b) SEM image of Mg nanoparticles synthesized by the HPMR method (reproduced with permission from [50, 53]).

Figure 4 compares the observation results from TEM and SEM techniques. The similar information obtained by SEM and TEM methods is the size and shape of the Mg nanoparticles. After hydrogen plasma synthesis, the obtained Mg nanoparticles have a size from tens of nm to a few hundred nm and the average size is around 300–500 nm. Most of the Mg particles have a hexagonal structure. The main difference lies in the dimensional information. TEM provides a 2-dimensional image, while the SEM may provide a 3-dimensional one.

**Figure 5:**
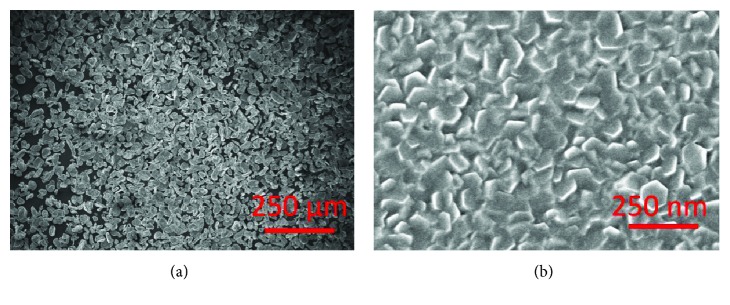
SEM images of (a) 325 mesh Mg particles from Alfa Aesar and (b) top view of the Mg thin film on a glass substrate (reproduced with permission from [50]).

Figure 5 presents the SEM images of two Mg samples.  Figure 5(a) shows commercialized Mg particles with a mean size of 40–50 *μ*m.  Figure 5(b) shows the Mg thin film deposited on a glass substrate by a direct current magnetron sputtering technique. The crystallite size of Mg on the film surface is around 50–100 nm. From the SEM observation in Figure 5(b), the shape of the small crystal domain is a hexagonal structure, which means that the thin-film synthesis is along the c-axis since the basal plane (0001) of a hexagonal close-packed Mg has the minimum surface energy [54]. Together with the Mg nanoparticles in Figure 4, we may see that the Mg samples by different synthesis methods may present quite a different morphology although both have a hexagonal structure. The SEM technique offers the possibility to observe the surface morphology of the samples.

### 2.3. Phase and Composition Determination

**Figure 6:**
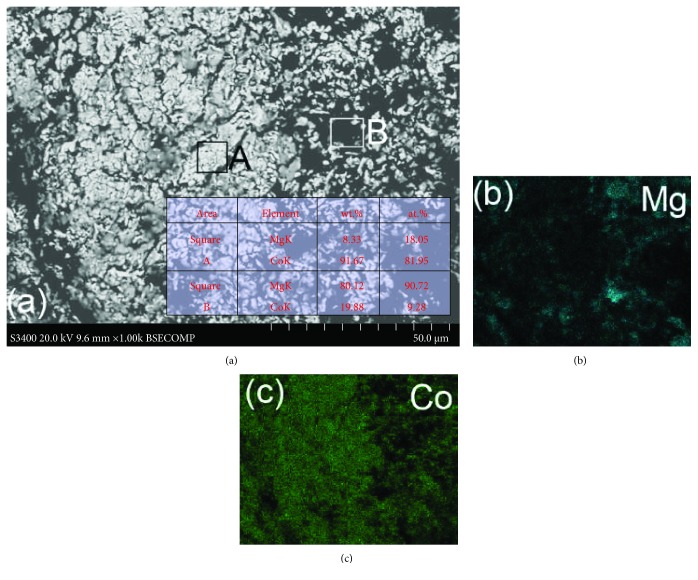
(a) BSE-SEM image of the Mg_50_Co_50_ sample milled for 0.5 h and the EDS mapping of the Mg element (b) and Co element (c). The inserted table provides the EDS quantitative results of elemental analysis in the selected square areas of A and B in (a) (reproduced with permission from [49]).

Figure 6 shows the result of the SEM observation and elemental analysis of the Mg_50_Co_50_ sample milled for just 0.5 h. Milling the mixture sample for 100 h results in the formation of the Mg_50_Co_50_ alloy with a bcc structure. After milling for only 0.5 h, it has mainly two Mg and Co phases, which can be confirmed from EDS mapping and X-ray diffraction measurements [49]. When we combine the EDS mapping results with the SEM image, we can see that the small white color particles are Co and the large dark ones are Mg. Here it may demonstrate that through the combination of SE-SEM and BSE-SEM observations as well as EDS elemental analysis mapping and X-ray diffraction techniques, a comprehensive understanding of the phase and composition of the samples may be acquired.

**Figure 7:**
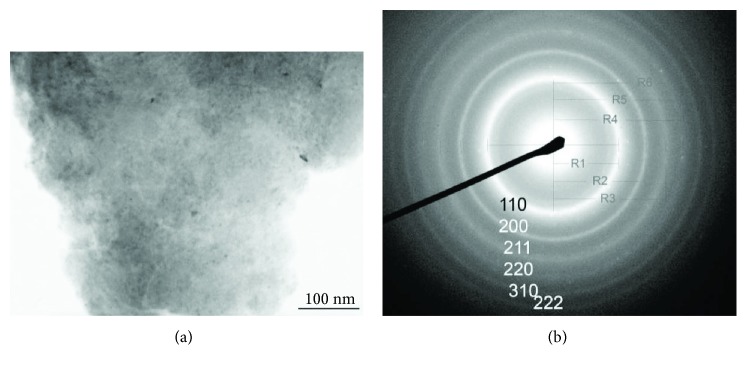
(a) Bright-field TEM image and (b) indexed electron diffraction pattern of the Mg_50_Co_50_ alloy with a bcc structure synthesized by ball milling for 100 h (reproduced with permission from [51]).

Figure 7 presents the bright-field TEM image and electron diffraction of the bcc-structured Mg_50_Co_50_ alloy. In Figure 2, we already discussed that this bcc-structured Mg_50_Co_50_ alloy is uniform in size with a particle size of a few *μ*m and a crystallite size of a few nm. The XRD result shows a set of well-broadened reflection peaks [21, 51] and from the broadening, the crystallite size is calculated to be 1–5 nm. In this size, there is no clear boundary between the nanocrystallite and amorphous state, and it is difficult to determine the local lattice structure based on the broadened reflection peaks. However, the electron diffraction characterization attached on the TEM equipment may provide some key information about the lattice structure of the Mg_50_Co_50_ alloy after milling for 100 h. After we measured the radii of the electron diffraction rings in Figure 7(b), it was found that the radii (*R*_1_ to *R*_6_) of the diffraction rings from the inside to the outside agree well with the rule of (1).

(1) $$\begin{array}{c} {R_{1}}^{2}:{R_{2}}^{2}:{R_{3}}^{2}:{R_{4}}^{2}:{R_{5}}^{2}:{R_{6}}^{2}=1:2:3:4:5:6. \end{array}$$

This unique accordance indicates that the Mg_50_Co_50_ alloy is well indexed as a bcc structure. Here we may see that sometimes, electron diffraction measurements along with SEM may provide a key local lattice structure of the samples, especially when the samples are at a scale of several nm.

**Figure 8:**
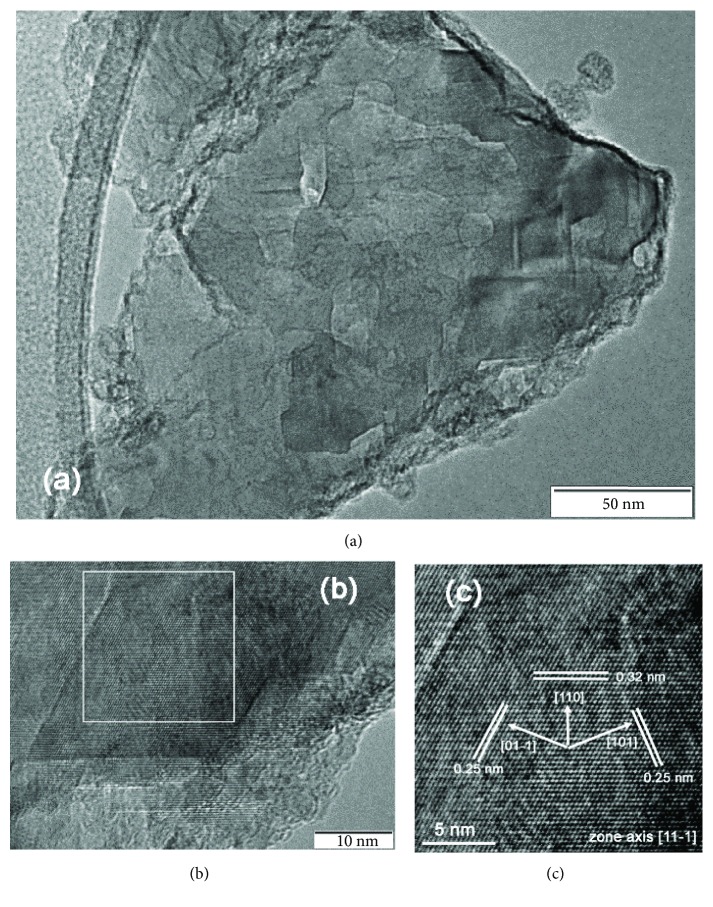
TEM images of (a) a typical MgH_2_ particle in the catalyzed MgH_2_ nanocrystallite sample, (b) a typical MgH_2_ nanocrystal area, and (c) a lattice spacing and orientation indexed area marked in (b) (reproduced with permission [20]).

Figure 8 presents TEM images of a typical MgH_2_ polycrystalline particle in the MgH_2_ nanocrystallite sample by a homogeneously catalyzed solution synthesis [20, 55, 56]. This particle has a size of 200–300 nm and some catalyzed Ti with an amorphous-like phase or a crystalline state is confirmed to be located mainly on the rims of the MgH_2_ nanocrystals.  Figure 8(b) shows one MgH_2_ domain with a size of about 50 nm. Since the observation of the TEM images is under a high quality vacuum, it is necessary to prove that the observed area is MgH_2_ and not Mg due to evacuation or MgO from oxidization. This key information can be provided from the indexed lattice fringes in Figure 8(c). The three spacings in Figure 8(c) of 0.25, 0.25, and 0.32 nm are in good agreement with the distances of (01-1), (110), and (101) planes of the MgH_2_ phase, respectively. This also indicates the electron beam parallel to the (11-1) zone axis. Here we can see that HR-TEM observation and lattice fringe indexation may provide some key information for phase determination in the selected area.

### 2.4. Understanding the Formation Mechanism

**Figure 9:**
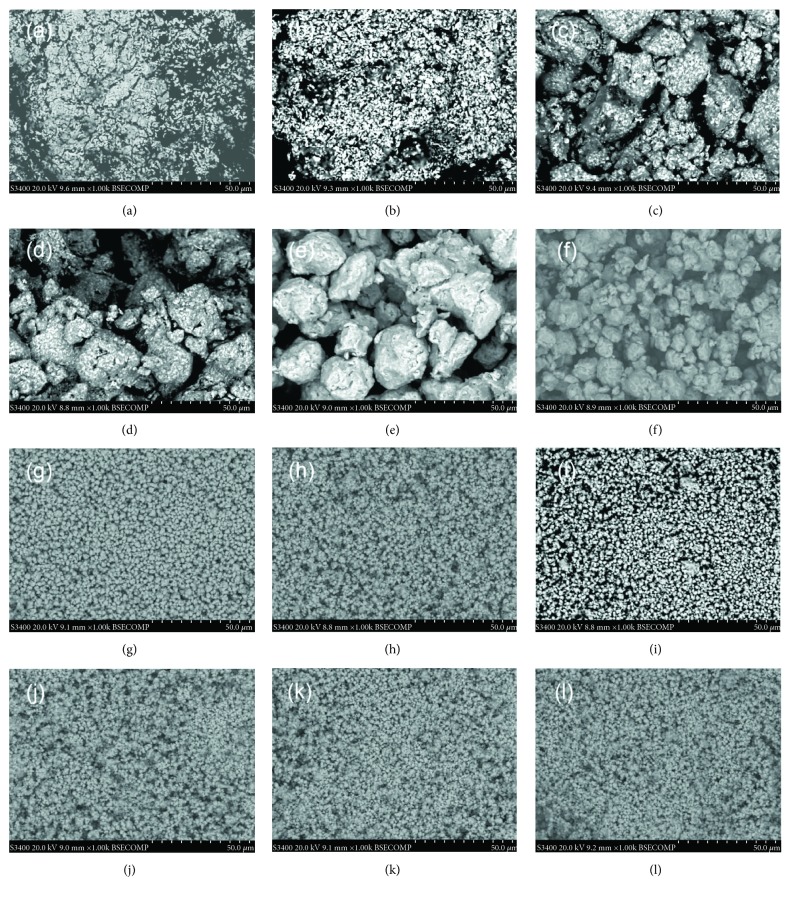
BSE-SEM images (at a magnification of 1000) of the Mg_50_Co_50_ samples milled at the following durations: (a) 0.5 h, (b) 2 h, (c) 5 h, (d) 10 h, (e) 25 h, (f) 40 h, (g) 50 h, (h) 75 h, (i) 100 h, (j) 200 h, (k) 300 h, and (l) 400 h (reproduced with permission from [49]).

**Figure 10:**
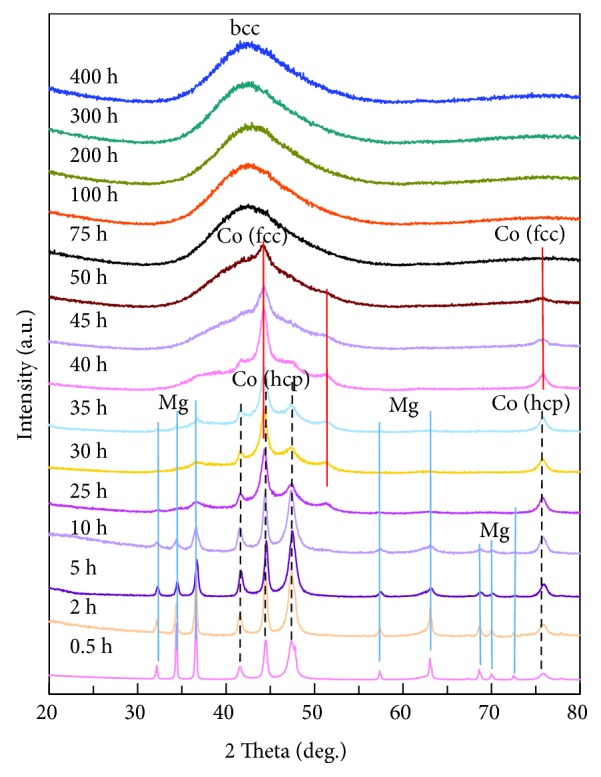
XRD patterns of the Mg_50_Co_50_ samples ball milled for various durations (0.5 to 400 h) (reproduced with permission from [49]).

XRD may provide the phase and composition of the samples. SEM may present morphology information of the samples, especially on the surface of the particles. The combination of SEM and XRD may be very helpful to the understanding of the evolution mechanism during the synthesis and formation process. Figures 9 and 10 present the BSE-SEM images and XRD patterns of the Mg_50_Co_50_ samples milled for various durations from 0.5 to 400 h [49, 51]. From the XRD curves, we may clarify the formation mechanism of the phases in the Mg_50_Co_50_ samples milled from 0.5 to 400 h, as in (2) (hcp means hexagonal close packed and fcc means face-centered cubic).

(2) $$\begin{array}{c} Mg+Co‐hcp\to {Mg}_{nano}+{Co}_{nano}‐hcp\to {Mg}_{nano}+{Co}_{nano}‐hcp+{Co}_{nano}‐fcc\to bcc+{Co}_{nano}‐fcc\to bcc. \end{array}$$

With the combination of the SEM in Figure 9 and the XRD results, the phase and morphology evolution process during the milling of Mg_50_Co_50_ samples from 0.5 to 400 h can be summarized as follows: firstly, with the beginning of the ball milling, small Co particles are well dispersed on the big Mg particles; secondly, large Mg particles are cracked into small ones and Co particles stick on the surface of Mg particles; thirdly, after around 25 h of milling, the Co phase with an fcc structure is formed and Co particles dissolve into the Mg ones; fourthly, particle sizes continue to decrease and the bcc phase appears after 45 h of milling; and fifthly, after 100 h, only the bcc phase with a crystallite size of only a few nm remains and the particles of the Mg_50_Co_50_ samples change from irregular shapes to round ones from further welding during the milling. The combination of SEM observation and XRD phase identification may offer an important solution to the understanding of the formation evolution mechanisms.

### 2.5. Correlation Study of Properties and Microstructure

**Figure 11:**
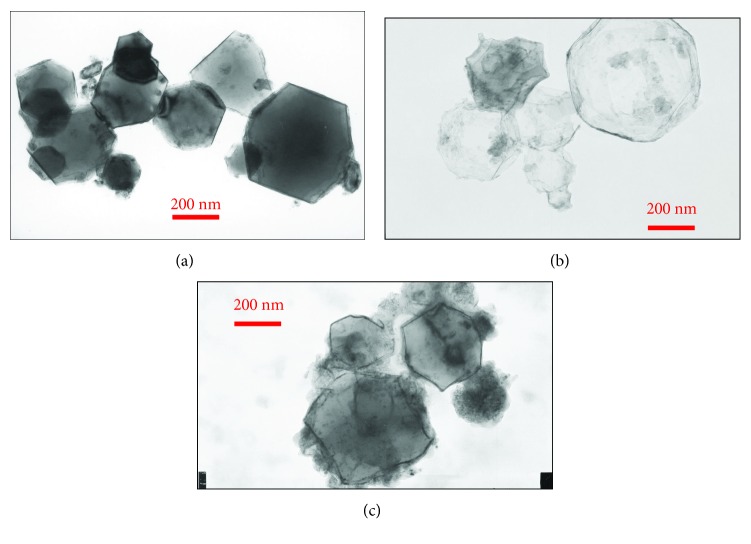
TEM images of (a) Mg nanoparticles synthesized by a hydrogen plasma metal reaction method, (b) MgH_2_ nanoparticles after hydrogenation of the Mg sample in (a), and (c) the Mg nanoparticle sample after hydrogen absorption and desorption steps (reproduced with permission [53]).

**Figure 12:**
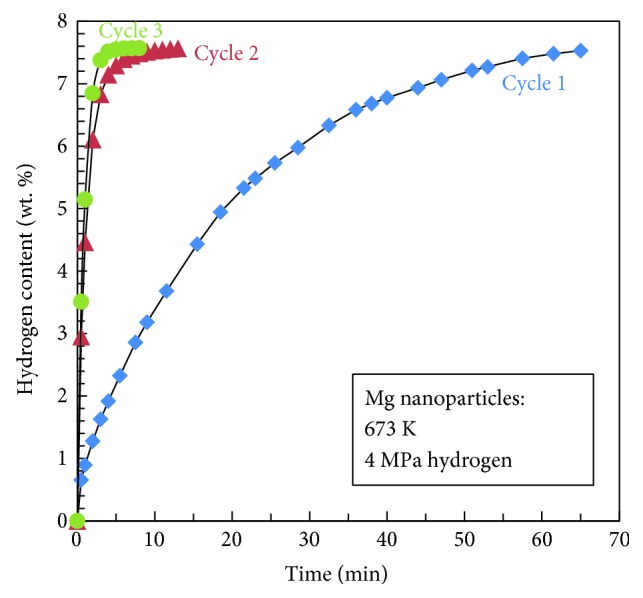
The first three hydrogen absorption curves in 4 MPa hydrogen atmosphere and at 673 K for Mg nanoparticles prepared by the HPMR method (reproduced with permission [53]).

It is well known that the physical and chemical properties of samples can be greatly affected by the microstructure of the samples including shape, particle size, size distribution, and surface morphology, which can be well evaluated by TEM and SEM techniques.  Figure 11 presents the TEM images of the Mg nanoparticles before and after hydrogen absorption and desorption processes.  Figure 12 demonstrates the first and second cycles of the hydrogen absorption kinetics of Mg nanoparticles. We have already discussed the microstructure of Mg nanoparticles as prepared by the HPMR method [8, 50, 53]. In Figure 11(b), we may see that the hydrogenated sample, MgH_2_ (Mg hydride), has a very light and transparent feature compared to the Mg nanoparticles. This is due to the fact that Mg nanoparticles in Figure 11(a) are in a metal state, while the MgH_2_ nanoparticles have a semiconductor feature. This difference makes the two samples show different optical characteristics under an electron beam. Actually, this phenomenon is applied for the research and development of metal hydride-based switchable mirrors [57–60]. Besides this difference, when we compare the as-prepared Mg nanoparticles (Figure 11(a)) with the Mg nanoparticles after hydrogen absorption and desorption cycles (Figure 11(c)), we can see that the sample of Mg nanoparticles after hydrogen storage cycles show broken surfaces and some sponge-like particles. This is because the particle surfaces are cracked due to the entry and exit of hydrogen atoms during the hydrogen absorption and desorption processes. This morphology difference is thought to play an important role in the hydrogen storage properties of the samples.

In Figure 12, we can see from the hydrogen absorption curves that the Mg nanoparticle sample by the HPMR method at the first cycle absorbs 7.53 wt.% hydrogen in 65 min, while it absorbs almost the same amount of hydrogen at the second and third cycles only after less than 15 min absorption. The Mg nanoparticles had not been subjected to the activation procedure before the three absorption cycles discussed in the previous line: this testifies to the superior hydrogen absorption properties of the nanoparticles prepared by the HPMR method. When we combine the TEM observation results and the ones with the hydrogen absorption kinetics together, we may see that the microstructure change on the surface of the Mg nanoparticles significantly influences the hydrogen absorption properties of the samples. This demonstrates that advanced TEM and SEM microscopy techniques may play essential roles in clarifying relationships between the properties and microstructures of the materials.

### 2.6. In Situ Observation of Hydrogen Reaction Mechanism

**Figure 13:**
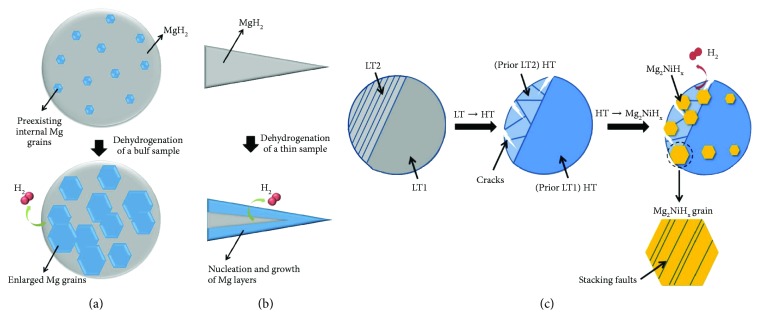
Schematic of hydrogen release mechanisms from (a) bulk MgH_2_, (b) thin MgH_2_, and (c) bulk Mg_2_NiH_4_ samples (reproduced with permission from [61, 62] (under the Creative Commons Attribution License/public domain)).

In situ scanning techniques are very helpful to understand the hydrogenation and dehydrogenation reactions of Mg-based materials. As a result, direct observations could be obtained to strongly support other experiments such as DSC and TG-MS. Nogita et al. [61] studied the dehydrogenation mechanism of MgH_2_ in different sizes by in situ TEM. They reported that the hydrogen release mechanism from bulk MgH_2_ with a particle size of 2 *μ*m was based on the growth of the multiple preexisting Mg grains (crystallite within the MgH_2_ matrix in Figure 13(a)) present, which was due to the difficulty of fully transforming all of the Mg during a hydrogenation cycle. On the other hand, in thin samples analogous to nanopowders, dehydrogenation occurs by a “shrinking core” mechanism as shown in Figure 13(b). In the case of Mg_2_NiH_4_, Tran et al. [62] studied the dehydrogenation mechanism of bulk Mg_2_NiH_4_ using in situ TEM. It was found that the dehydrogenation was based on a mechanism of the nucleation and growth of Mg_2_NiH_*x*_ (*x*~0–0.3) solid solution grains and was greatly enhanced in the presence of crystal defects occurring as a result of the polymorphic phase transformation as shown in Figure 13(c). Also importantly, with atomic resolution TEM imaging, a high density of stacking faults is identified in the dehydrogenated Mg_2_NiH_*x*_ (*x*~0–0.3) lattices. Zhu et al. [46] adopted the method of hydriding chemical vapor deposition to synthesize a single-crystal MgH_2_ nanofiber. Then, the phase change of the as-obtained MgH_2_ nanofiber in the desorption process was observed with the in situ TEM. The results indicated that the orientation relationship between MgH_2_ and Mg during the phase change was one of the zone axis of MgH_2_ (110) parallel to the Mg (0001) zone axis, or one of the plane (110) of MgH_2_ parallel to the basal plane of Mg (0001). Similar results were reported by Paik et al. [43].

Besides the pure MgH_2_ and bulk Mg_2_NiH_4_, the catalysts were also introduced into the MgH_2_ to investigate the microstructural changes by in situ techniques in the dehydrogenation process. Isobe et al. [42] ball milled the MgH_2_ with different amounts of Nb_2_O_5_ to study the effects of Nb_2_O_5_ on the desorption of MgH_2_ by in situ TEM. It was found that the MgH_2_ doped by 1 mol% Nb_2_O_5_ started at 150°C and then the nanosized Mg was formed, while the desorption of MgH_2_ catalyzed with 10 mol% started at the interface between MgH_2_ and Nb_2_O_5_ with the temperature increasing, which suggested that hydrogen atoms could diffuse from the MgH_2_ phase to the interface between the MgH_2_ and Nb_2_O_5_. Similarly, Kim et al. [44] also prepared the NbF_5_-doped MgH_2_ by ball milling and then studied the microstructural changes in the desorption process of the as-prepared sample by in situ TEM. Results showed that the amorphous Nb-F thin layer contacted the MgH_2_ phase at 25°C, while the amorphous Nb-F layer was transformed to a metallic Nb crystalline layer after complete desorption at 250°C. Based on the above analysis, the in situ technique allows researchers to clearly study the samples in real time under special conditions (such as by heating or at a certain atmosphere), which may strongly support other analyses. In a word, the in situ technique is a powerful strategy to grasp more information from the samples than traditional microscopy.

## 3. Summary and Prospects

The utilization of SEM and TEM techniques for characterizing the size, morphology observation, phase and composition determination, and formation and reaction mechanisms in Mg-based hydrogen storage materials fabricated by different nanoprocessing methods are presented in this paper. Although the information obtained by SEM and TEM about the size and shape of Mg-based materials are similar, TEM provides a 2D image of the particles while SEM may obtain a 3D one. Using the TEM measurement, we could easily obtain the size, size distribution, and average size of various Mg-based samples. The SEM technique also offers the possibility to observe the surface morphology. Additionally, we may well understand the phase, composition, and structure and formation mechanism of Mg-based hydrogen storage materials by the combination of different techniques. However, conventional electron microscopies have challenges compared with in situ scanning techniques when studying the hydrogen reaction mechanism. A special sample holder which may transfer samples from the glove box to the TEM/SEM equipment presents great advantages when it comes to observing sensitive samples. In situ electron microscopy can be allowed to study some changes of the Mg-based materials in real time under special conditions and in turn directly verify the hydrogen reaction mechanism. As modern science moves from studies of structure and ground states to dynamics and functionalities, electron microscopy will clearly experience a revolutionary growth in capabilities in the next decade, from ultrasmall to ultrafast and to multidimensions, which could result in transformative advances in many fields of science and engineering [63].
